# ESOT Roadmap for Advanced Therapy Medicinal Products in Transplantation: Navigating Regulatory Challenges to Enhance Access and Care

**DOI:** 10.3389/ti.2024.13485

**Published:** 2024-10-14

**Authors:** Ekaterine Berishvili, Lorenzo Piemonti, Eelco J. P. de Koning, Sandra Lindstedt, Hanne Scholz, William E. Scott, Celine Auxenfans, Paul Johnson, Dominique E. Martin, Penilla Gunther, Devi Mey, Luciano Potena, Olivier Thaunat

**Affiliations:** ^1^ Cell Isolation and Transplantation Centre, Department of Surgery, Geneva University Hospitals, Laboratory of Tissue Engineering and Organ Regeneration, Department of Surgery, University of Geneva, Geneva, Switzerland; ^2^ Diabetes Research Institute, IVita-Salute San Raffaele University, Milan, Italy; ^3^ Department of Internal Medicine, Leiden University Medical Center, Leiden, Netherlands; ^4^ Department of Clinical Sciences, Lund University, Lund, Sweden; ^5^ Lund Stem Cell Center, Lund University, Lund, Sweden; ^6^ Wallenberg Center for Molecular Medicine, Lund University, Lund, Sweden; ^7^ Department of Cardiothoracic Surgery and Transplantation, Skåne University Hospital, Lund, Sweden; ^8^ Hybrid Technology Hub Centre of Excellence, Institute of Basic Medical Science, University of Oslo, Oslo, Norway; ^9^ Regenerative Medicine, Stem Cells, and Transplantation Theme, Translational and Clinical Research Institute, Newcastle University, Newcastle upon Tyne, United Kingdom; ^10^ Banque de Tissus et de Cellules des Hospices Civils de Lyon, Edouard Herriot Hospital, Lyon, France; ^11^ Oxford Consortium for Islet Transplantation, Oxford Centre for Diabetes, Endocrinology, and Metabolism (OCDEM), Churchill Hospital, University of Oxford, Oxford, United Kingdom; ^12^ School of Medicine, Faculty of Health, Deakin University, Waurn Ponds, VIC, Australia; ^13^ The Swedish Parliament, Stockholm, Sweden; ^14^ The European Society for Organ Transplantation, Amsterdam, Netherlands; ^15^ Istituto di Ricovero e Cura a Carattere Scientifico (Scientific Institute for Research, Hospitalization, and Healthcare) (IRCCS) University Hospital of Bologna Sant Orsola Polyclinic, Bologna, Italy; ^16^ Centre International de Recherche en Infectiologie (CIRI), Université de Lyon, Inserm, U1111, Université Claude Bernard Lyon 1, CNRS, UMR5308, ENS de Lyon, Lyon, France

**Keywords:** organ transplantation, cell transplantation, advanced therapy medicinal products (ATMPs), regulatory challenges, patient access

## Abstract

The field of organ transplantation is experiencing a transformative shift with the rise of Advanced Therapy Medicinal Products (ATMPs), which include gene therapies, somatic cell therapies, and tissue-engineered products. These therapies offer new, potentially curative treatments for longstanding medical challenges, impacting numerous patients. However, their adoption is hindered by complex regulatory frameworks, high production costs, and inconsistent access across Europe. The ESOT ATMP Task Force’s position paper analyzes these challenges from research to clinical application, advocating for a coordinated strategy to position Europe as a leader in ATMP development. It proposes specific actions such as streamlining regulatory pathways to accelerate approvals, boosting funding for ATMP research, and creating specialized facilities for development and implementation. The paper also highlights the critical roles of patient engagement and real-world evidence in optimizing clinical and regulatory practices.

## Introduction

Advanced Therapy Medicinal Products (ATMPs) mark a transformative shift in the field of organ transplantation. These products, also known as “Biologics,” are produced through complex biological processes involving living cells, tissues, or genetic materials. The development of ATMPs in organ transplantation aims to address critical issues such as organ scarcity, graft rejection, and the long-term viability of transplant outcomes. In the European Union, ATMPs fall under the regulatory framework for biological products, specifically Directive 2001/83/EC and Regulation EC/1394/2000. Within the EU, ATMPs are categorized into four main groups: gene therapy, somatic cell therapy, tissue-engineered therapies, and combined advanced therapies.

Despite their significant potential, ATMPs face numerous obstacles that hinder their practical application in clinical settings. One of the primary obstacles is the complex and lengthy regulatory approval process for ATMPs. The European Medicines Agency (EMA) and national competent authorities have established stringent guidelines to ensure the safety and efficacy of these innovative therapies. However, navigating these regulatory requirements can be time-consuming and resource-intensive, particularly for academia that lack the necessary expertise and infrastructure. Furthermore, high manufacturing costs associated with ATMP development and production, and significant economic hurdles after product approval, all of which impede the progress of ATMPs from research to clinical application and consequently limit patient access to these innovative therapies.

This position paper by the ESOT ATMP Task Force provides a detailed analysis of the multifaceted challenges that arise from the research phase through to the clinical implementation of ATMPs in organ transplantation across Europe. It identifies key hurdles in the development and deployment of these therapies and suggests strategic recommendations to facilitate their integration into healthcare systems. This would subsequently broaden the accessibility of ATMPs to patients across Europe, significantly enhancing outcomes in transplantation medicine.

## Promises of ATMPs in Transplantation

### Organ Shortage

The demand for organ transplants far exceeds the available supply, leading to significant morbidity and mortality among patients requiring transplantation. ATMPs, including tissue engineering and xenotransplantation, represent promising solutions in regenerative medicine. These technologies offer potential breakthroughs in addressing significant medical challenges by repairing or replacing damaged tissues and organs. Tissue engineering aims to create functional organ substitutes or enhance the body’s innate regenerative capabilities, potentially alleviating the demand for donor organs [1, 2]. Xenotransplantation, involving the transplantation of organs or tissues from one species to another, has been explored to address organ scarcity; notably from genetically modified pigs to humans in recent years [3].

Tissue engineering approaches, such as biodegradable scaffolds loaded with cells to create functional replacements for damaged tissues are being explored to address the limited availability of donor organs and tissues [4–8]. These methods leverage the body’s natural regenerative capabilities to provide personalized and biocompatible solutions for patients requiring functional organ or tissue replacement.

### Improvement of Graft Quality/Regeneration

Recent advancements in *ex vivo* organ perfusion (EVOP) have positioned it as a promising platform for organ-specific gene and cell therapy in transplantation [9, 10]. EVOP systems allow for precise genetic modifications to organs, delivery of gene therapies to the perfused organ, making it possible to correct genetic defects or enhance the organ’s resilience against ischemia-reperfusion injury, a common post-transplant complication. Importantly, adenoviral vectors used during EVOP have been shown to reduce inflammation associated with gene delivery, as demonstrated in pig models of lung transplantation [11, 12]. This finding suggests that gene therapy can be effectively integrated into organ perfusion processes without exacerbating inflammatory responses, thus improving transplant outcomes. Moreover, the controlled environment of EVOP reduces the risk of off-target effects, increasing the safety of gene therapy. While EVOP’s ability to assess and preserve organs remains significant, its role in enabling targeted gene delivery is particularly relevant for advancing transplantation outcomes and expanding the available donor pool by potentially rescuing marginal organs [9, 10, 13–16].

Somatic cell therapies involve manipulating cells or tissues to modify their biological characteristics. These therapies span a range of applications, from treating acute injuries to chronic diseases, and are particularly relevant for regenerating or repairing damaged tissues; potentially negating the need for complete organ replacement. Developments in stem cell research enhance the potential of somatic cell therapies, allowing for patient-specific stem cells to be used in various medical applications, including drug testing and regenerative medicine [17, 18].

### Graft Rejection and Long-Term Outcomes

The recipient’s immune system rejecting the transplanted organ remains a major hurdle in organ transplantation. It largely explains the stagnation of graft half-life over the last decades [19], thereby contributing to organ shortage.

Current prevention strategies predominantly involve immunosuppressive therapy, which poses significant side effects and long-term complications, including increased susceptibility to infections and cancer. ATMPs such as Chimeric Antigen Receptor (CAR) T-cell therapy and regulatory T-cell (Treg) therapy offer innovative approaches to mitigate these risks. CAR T-cell therapy, a type of gene therapy, has proven effective in treating post-transplant lymphoproliferative disorders, a common complication in solid organ transplants, by allowing genetically engineered T-cells to target and eliminate cancerous cells [20]. Treg therapy aims to induce immune tolerance, potentially reducing the dependency on immunosuppressive treatments [16, 21–23]. Moreover, the development of immune cell phenotype modifications, such as CAR T, CAR Treg, and CAR B cells, is advancing transplant immunology by enhancing the specificity and efficacy of immunosuppression. CAR Treg therapy merges the targeting capabilities of CAR T-cell therapy with the regulatory properties of Tregs, providing a dual benefit in organ rejection prevention. Emerging CAR B-cell therapy may further enhance post-transplant immune regulation. Mesenchymal stromal cells (MSCs) offer an alternative cell-based approach for enhancing transplant outcomes by modulating immune responses and promoting tissue repair. Early-phase clinical trials have demonstrated the safety and feasibility of MSC infusions in kidney and liver transplant recipients. MSCs can potentially reduce the reliance on immunosuppressive medications by secreting anti-inflammatory cytokines and promoting graft tolerance. This approach aims to improve graft survival and decrease the side effects associated with long-term immunosuppression [24–26].

The longevity and functionality of transplanted organs are critical for patient outcomes. Chronic rejection, characterized by the gradual loss of organ function, remains a leading cause of transplant failure. ATMPs, through their ability to engineer organs with enhanced biocompatibility or to modulate the immune response more precisely, hold the promise of extending the life and function of transplanted organs. Tissue-engineered organs using the recipient’s own cells combined with approaches that repair, or regenerate organ tissues could decrease rejection risks and enhance both functionality and longevity [1, 2].

Gene therapy could be used to modify the genetic material of donor organs *ex situ* to decrease rejection likelihood or induce recipient tolerance. By producing immunomodulatory proteins within donor grafts, gene therapy can achieve localized immunosuppression or even donor-specific tolerance, thus possibly eliminating the need for general systemic immunosuppression [27].

## Accessibility to Approved ATMPs Across EU and Reimbursement Challenges

The integration of ATMPs into clinical practice heralds a new era in transplantation medicine, where it may be possible to tailor treatments to the individual patient’s needs, improving quality of life and longevity. However, this innovation comes with the requirement for rigorous evaluation to ensure safety, efficacy, and ethical considerations; underscoring the importance of robust regulatory frameworks and clinical guidelines to manage their application in transplantation. Due to the broad scope of applications, navigating the journey from research to market for ATMPs presents a complex and formidable challenge. The development and commercialization process is not only lengthy and expensive but also carries a high risk of failure. Following approval, these therapies often encounter what is termed the “economic valley of death,” a phase where financial constraints significantly restrict patient access to these treatments. This issue is pervasive across medical fields, signalling a widespread systemic challenge.

Academic institutions and charities, which have been instrumental in pioneering ATMP research, frequently lack the financial capacity to support costly clinical trials. This financial gap often necessitates forming partnerships with corporations or establishing biotech startups to secure the necessary funding for continued development. The production costs associated with ATMPs, such as viral vectors and cell products, are substantial, sometimes reaching several million euros per patient. These costs are driven by stringent safety and quality standards coupled with the treatment of conditions that range from relatively common to extremely rare, which limits the patient base and increases unit costs.

Consequently, the high costs of these therapies result in prolonged or unsuccessful negotiations with health systems, leading to significant variations in access to approved ATMPs across Europe, driven by diverse reimbursement systems and policies [28]. Germany widely reimburses ATMPs through its public health system, whereas Ireland does not cover several EMA-approved ATMPs. The UK, France, and Spain provide more limited reimbursement, restricted to specific clinical indications. Such economic pressures have led to the withdrawal from the European market of several efficacious and approved products; depriving patients of potentially life-saving treatments. Bluebird bio withdrew Skysona and Zynteglo in 2022 despite their efficacy, citing non-viable reimbursement negotiations.^1^

Furthermore, despite considerable industry efforts to broaden access to autologous CAR T-cell therapy, numerous barriers persist, including complex logistics involving intercontinental cell shipments, manufacturing slot reservations, and bureaucratic delays.^2^ A 2020 study on the accessibility of CAR T-cell therapy for patients with diffuse large B-cell lymphoma in Germany, France, Italy, and Spain showed that a significant proportion of patients, 58%–83% within the EMA-approved label population and 29%–71% deemed clinically eligible, did not receive commercially approved CAR T-cell products.^3^ In Spain, it takes about 18 months from EMA approval to the authorization of price and reimbursement for orphan drugs, which include most ATMPs. Nearly one-third of these approved orphan drugs fail to secure reimbursement, with half being the only available treatment options for their respective diseases.^4^ As of May 2023, only 20% of EMA-approved ATMPs were reimbursed by Spain’s public national health system.^5^ These reimbursement challenges are likely driven by cost implications.

In some cases, companies discontinue an ATMP post-approval if the economic returns do not justify the investment. For instance, UniQure discontinued Glybera in 2017 after treating only one patient, as the costs and limited use did not justify the investment: even with a price tag of one million euros per patient. Similarly, Strimvelis^®^, initially developed by GSK and later acquired by Orchard Therapeutics, was discontinued due to commercial viability issues. Additionally, financial withdrawal by Valline Holding Srl led to the cessation of Holoclar^®^, the first stem cell derived ATMP in Europe, highlighting the precarious nature of sustaining such innovative treatments in the European market [1].

Systemic challenges in the European sector have led to the discontinuation of ATMPs, thereby jeopardizing access to life-saving treatments for a considerable number of citizens. Reimbursement constraints, insufficient economic returns, and bureaucratic hurdles have impeded the sustained development and commercialization of these therapies. Additionally, the high costs associated with the development and approval process deter many promising treatments from reaching the market. While Europe has historically been a leader in developing ATMPs, it has recently been outpaced by the US and Asia in advancements within this field. From 2014 to 2021, the number of ATMP clinical trials in the US and Asia-Pacific grew by 70% and 67%, respectively, while Europe’s growth remained stagnant.^6^ The US now hosts twice as many ATMP trials as Europe, and China nearly three times as many. Furthermore, the US dominates in the number of companies developing ATMPs and their contributions to the global new drug pipeline, with China’s growth in this area increasing by 456% between 2016 and 2021.

Despite Europe’s strong academic output in ATMP research, it falls short in translating this into practical therapies, contrasting sharply with the US and Asia where regulatory approvals are faster and the market pricing for ATMPs tends to be higher. Europe also struggles with a siloed approach to policymaking and has not capitalized on opportunities to foster growth in ATMP clusters, leaving it less competitive in attracting clinical trials and housing only 50% of the world’s ATMP manufacturing facilities compared to Asia’s rapid rise.^7^

This discrepancy poses a critical decision for the EU: to either remain a consumer of high-cost therapies developed abroad, potentially limiting patient access due to affordability issues, or to actively participate in the development of these therapies. To regain its footing, Europe should enhance funding, streamline regulatory processes, and strategically support ATMP innovation hubs.

## ESOT ATMP Taskforce Position

Europe’s ambition to emerge as a leader in the field of ATMPs hinges on its ability to increase funding, streamline regulatory processes, and strategically foster ATMP-innovation hubs to expedite clinical development. Recognizing the challenges associated with ATMPs—from the bench to bedside—the European Society for Organ Transplantation (ESOT) has convened several meetings to underscore the need for efficient procedures that facilitate the assessment, access, and clinical integration of these therapies before they become widely available in the market. This proactive approach aims to eliminate access barriers and streamline the journey from experimental phases to clinical application.

ESOT strongly advocates for refining the regulatory framework to ensure quick access to ATMPs without compromising on their safety and efficacy. The society promotes a holistic strategy addressing regulatory, manufacturing, cost, and healthcare system integration challenges. A collaborative approach involving stakeholders and utilizing evidence-based methods is essential to enhance the regulatory framework, enabling timely access to ATMPs while upholding rigorous safety and efficacy standards.

In support of this initiative, ESOT has established a task force focused on advancing ATMPs, dedicated to improving patient access to these advanced therapies, managing regulatory and ethical changes, and promoting the continuous development of innovative healthcare solutions within the European Union.

The journey of ATMPs from experimental phases to clinical application is intricate and multifaceted. Academic institutions, typically at the forefront of developing these innovative therapies, have been compelled to conform to stricter pharmaceutical manufacturing and clinical trial standards. A critical element of the ATMP regulation is the HE clause, which allows individual Member States to authorize the use of ATMPs without centralized marketing under specific conditions, primarily for patients with unmet medical needs. This exemption not only provides patients with access to potentially life-saving therapies but also opens avenues for collecting real-world evidence (RWE). RWE, derived from real-world data (RWD) collected outside conventional clinical trial settings, provides invaluable insights into the effectiveness, safety, and practical application of ATMPs, including cell and gene therapies, in routine clinical practice. Integrating RWE into ATMP development can significantly streamline the process by providing evidence on long-term outcomes, patient quality-of-life, and comparative effectiveness. This integration facilitates a more informed and nuanced understanding of ATMPs’ real-world impact, aiding in the refinement and innovation of clinical trial designs and the development of more targeted and effective therapeutic interventions. However, concerns about the quality, consistency, and standardization of RWD must be addressed to ensure that the evidence generated is reliable and actionable. Efforts by regulatory bodies such as the EMA, particularly through initiatives like DARWIN EU^®^, are aimed at enhancing RWD quality and fitness for regulatory purposes by establishing standard methodologies and fostering data sharing across borders.^8^

The interpretation and implementation of HE varies widely across the EU, leading to inconsistencies in patient access and potential safety concerns. The issue of non-exportability of ATMPs under the HE poses another significant challenge, particularly since these treatments are intended for patients in therapeutic impasse. To address this, it is necessary to facilitate the exchange of ATMPs under HE between Member States, underscoring the importance of European mutual assistance. Furthermore, the coexistence of HE with the central marketing authorization route often leads to perceptions of unfair competition against commercially marketed medicinal products.^9^ ^,^ ^10^

The Committee on the Environment, Public Health, and Food Safety (ENVI) addressed some of these concerns by voting on 19 March 2024, on a set of 100 Compromise Amendments. These amendments not only preserve the Hospital Exemption within the Member States unchanged but also strengthen its implementation in the cross-border exchange of ATMPs, aiming to improve access and equity across Europe. In its current version, the Directive proposal does not introduce any change that could have a major impact on the management of manufacturing authorizations for ATMPs under hospital exemption. However, the modifications made by the Directive proposal suggest a strong willingness, through the collection of data, to reinforce the management of these products by the Member States and, through the communication with the EMA, to further harmonize the rules governing ATMPs under hospital exemption.

To this end, leveraging RWE becomes imperative. Recognizing the significance of this approach, the ESOT is well-positioned to lead the establishment and management of ATMP registries in the field of organ, cell, and tissue replacement. By doing so, ESOT can facilitate the systematic collection and analysis of RWD, thereby enriching the RWE pool. These efforts can contribute significantly to shaping the regulatory landscape, informing policy decisions, and guiding clinical practice in organ transplantation. The ESOT intends to require for standardized data collection methods to ensure the reliability and validity of the RWE generated. Furthermore, ESOT can play a pivotal role in fostering collaborations among scientific societies, transplantation centers, regulatory bodies, and the pharmaceutical industry to promote the sharing of RWD and RWE, thus driving forward the field of ATMPs.

The European legislation on ATMPs, which includes cell therapies, gene therapies, and tissue-engineered products, has significantly influenced academic networks involved in their development since its enactment approximately 20 years ago. This legislation, particularly Regulation (EC) No 1394/2007, reclassified cell and gene therapies that underwent “substantial manipulation” or were intended for non-homologous functions, moving them from the organ and tissue transplantation regulatory framework to that of pharmaceuticals. While this shift enhanced regulatory oversight and experience with ATMPs, it has affected approval rates due to the stringent and inflexible regulatory demands, significantly increased costs, and generated market uncertainty, which restrict investment from the pharmaceutical sector. Moreover, some holders of approved products have subsequently withdrawn them, primarily for regulatory and economic reasons, as discussed above.

Recently, the EMA released the second version of the draft guideline on quality, non-clinical, and clinical requirements for investigational ATMPs (EMA/CAT/123573/2024). This release marks a significant step in the review of the EMA’s pharmaceutical framework. The feedback from the initial draft underscored the necessity for clearer guidelines on non-viral and genome editing therapies and called for greater regulatory alignment, especially with FDA standards. The revisions improved terminology consistency and introduced a risk-based approach, yet stakeholders indicated that further refinements were needed, especially a clearer separation of guidelines for different ATMP types and more detailed guidance for their development. These ongoing legislative reforms and the discussions taking place across various platforms present an opportunity for ESOT to influence and help shape a regulatory framework that is both robust and conducive to the rapid development and integration of ATMPs and other emerging technologies in clinical practice. This framework should also establish clear guidelines for new and evolving therapeutic approaches, ensuring that such innovations are neither restricted by outdated regulations nor delayed when patient lives are at risk. This approach would enable the safe and effective delivery of promising therapies to patients across the EU, continuing the tradition of innovation in medical treatment.

## ESOT Recommendations

To streamline the development and accessibility of ATMPs in the field of transplantation in Europe, ESOT recommends implementing following strategies:

### Streamlining Regulatory Processes

The complexity and duration of the regulatory approval process can significantly impact the development and accessibility of ATMPs. It is essential for regulatory bodies to streamline their procedures to expedite the approval timeline and reduce associated costs, while still upholding stringent safety and efficacy standards. In revisiting the EMA/CAT definition of ATMPs, consideration should be given to whether minimally modified cell therapy products like the Stromal Vascular Fraction (SVF) warrant distinct regulatory pathways depending on their use in homologous versus non-homologous therapies. For example, the classification of SVF could potentially vary between applications in plastic surgery and treatments for scleroderma, focusing more on the nuances of the manufacturing process rather than just the clinical application. This approach would allow production facilities to use targeted risk assessments as a measure of manufacturing process quality, ensuring that regulatory standards are met without unnecessarily impeding product development and innovation.

### Increasing Funding for ATMP Research

Significant investment is required to develop ATMPs, yet public funding often overlooks these needs. National science funding should prioritize not only the creation of new knowledge but also the establishment of clinical trials and safety studies, activities typically classified at TRL4 and higher. Funding agencies are encouraged to increase their investments in ATMP research with a focus on academic research and development to facilitate scaling.

### Promoting Collaborations and Partnerships

Effective development and translation of ATMPs require collaboration among academic researchers, industry partners, and regulatory agencies. Increased support for partnerships, including funding, infrastructure, and regulatory support, is essential.

### Establishing Pre-ATMP Facilities

Developing pre-ATMP facilities can enhance the efficiency and success rate of ATMP projects by providing a platform for extensive testing of products for compatibility, safety, and efficacy before full-scale GMP production and for developing and validating quality control methods, particularly potency assays. This is critical for ensuring that promising therapies are not held back by a lack of foresight and experience by using non-compliant products in the formative research activities. Collaborative efforts to standardize potency assays for ATMPs with similar modes of action would streamline processes, save time, and facilitate easier comparisons between products.

### Establishing Centralized ATMP Facilities

Specialized facilities and expertise are crucial for the successful development and production of ATMPs. Centralized ATMP facilities would provide accessible infrastructure and regulatory affairs expertise, which are vital for clinical translation. Experts in these facilities should possess knowledge in establishing quality management systems, training personnel in Good Manufacturing Practices (GMP), defining release criteria, and navigating approval requirements.

### Enhancing the Efficiency and Accessibility of the Hospital Exemption (HE) Approval Pathway

HE pathway facilitates the use of ATMPs outside standard marketing authorization for patients with unmet medical needs. However, variations and inconsistent applications across countries reduce its effectiveness. Harmonizing HE rules is crucial to ensure that ATMPs, especially those not commercially viable, can still reach patients, support rapid manufacturing innovations, and provide uninterrupted treatment during clinical development.

### Optimizing ATMP Development in Europe Through Comprehensive Data Collection and Analysis

To assess the current status of ATMPs across Europe, it is critical to conduct a comprehensive survey that incorporates RWE derived from RWD collected beyond conventional clinical trials. RWE is essential for transitioning ATMPs from experimental phases to clinical use, providing deep insights into their effectiveness, safety, and impact on patient quality of life. These insights help improve clinical trial design and treatment efficacy.

To maximize the benefits of HE in ATMP development, it is imperative to establish robust registries. These registries should meticulously record patient outcomes, treatment specifics, and adverse events. The detailed data collected will facilitate the generation of RWE, enhancing the foundation for more informed regulatory and clinical decisions. This structured approach ensures that ATMPs are not only effective but also safe and well-suited to meet patient needs.

### Engaging With Patients and Patient Advocacy Groups

Incorporating the perspectives of patients and patient advocacy groups is crucial in the clinical development and translation of ATMPs. Engaging with these stakeholders early in the development process is essential for identifying and understanding the barriers and facilitators that could influence the adoption and impact of ATMPs on patient communities. Conducting empirical research on patient perspectives ensures that the clinical translation of ATMPs is responsibly aligned with the needs and expectations of patients. This collaborative approach can lead to more effective and patient-centered healthcare solutions.

## Conclusion

The field of organ transplantation, and more broadly, medicine, is undergoing a significant transformation driven by the development and integration of ATMPs. These innovative therapies are poised to revolutionize the treatment landscape by offering new, potentially curative options for conditions that have long challenged medical professionals and affected the lives of countless patients.

The ESOT ATMP task force has meticulously outlined the critical challenges and strategic recommendations in this position paper focused on the European context. As ATMPs continue to evolve, the key to harnessing their full potential lies in the effective collaboration among a diverse array of stakeholders. These include scientific societies such as ESOT, academic researchers who are often at the forefront of ATMP innovation, and pharmaceutical and biotechnological companies that facilitate the scaling and distribution of these therapies. Additionally, regulatory bodies play a pivotal role in ensuring that these therapies are both safe and effective, while patient groups contribute invaluable insights that help tailor ATMPs to meet actual patient needs and expectations.

**FIGURE 1:**
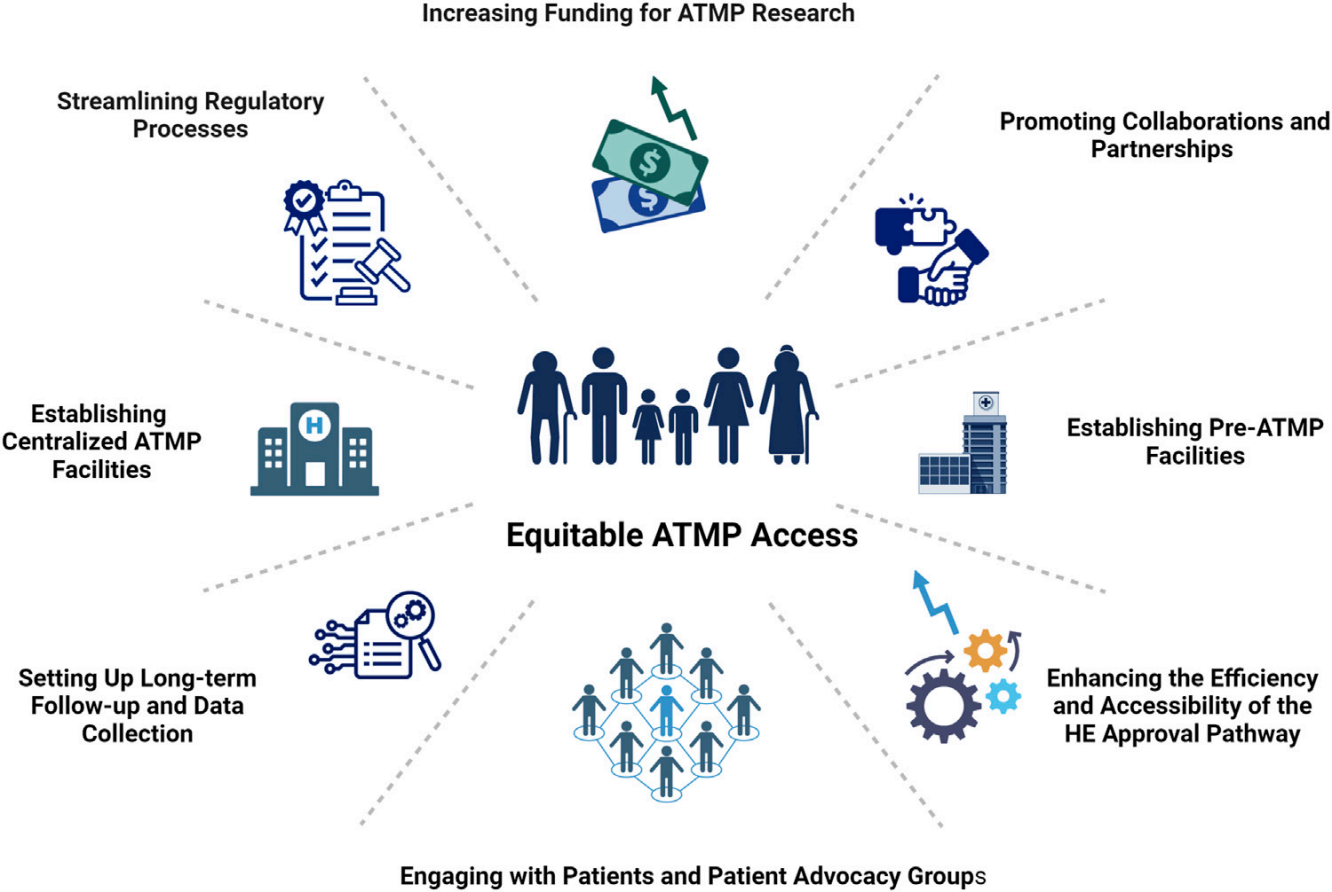
ESOT vision for accelerating equitable access to ATMPs. This figure outlines ESOT’s collaborative strategies to address translational barriers and facilitate the safe, rapid integration of ATMPs into clinical practice, promoting sustainable growth and equitable patient access to innovative medicines. Created in BioRender. Berishvili, E. (2024) BioRender.com/j50w273

The collective effort of these stakeholders is fundamental not only in overcoming regulatory, financial, and technical barriers but also in establishing a robust framework that supports the rapid development, approval, and integration of ATMPs into clinical practice (Figure 1). This collaborative approach ensures that the revolutionary potential of ATMPs can be realized, ultimately changing the course of diseases and significantly improving patient outcomes.

In essence, the transition of ATMPs from experimental therapies to mainstream treatment options represents a paradigm shift in medicine that holds promise for a future where many diseases may no longer be seen as terminal or incurable. As this field continues to advance, the continued engagement and coordination of all parties involved will be critical in making these life-altering therapies accessible to those who need them most.

## Data Availability

This position statement does not present original data. It is informed by a comprehensive review of current regulatory frameworks governing ATMPs in transplantation, relevant policy documents, and expert consensus within the ESOT task force.

## Footnotes

1
https://joppp.biomedcentral.com/articles/10.1186/s40545-021-00311-0

2
https://doi.org/10.1007/s40290-022-00428-w

3
https://doi.org/10.3389/fmed.2023.1128295

4
https://doi.org/10.1186/s13023-022-02610-4

5
https://doi.org/10.1016/j.stem.2023.07.004

6
https://www.efpia.eu/news-events/the-efpia-view/efpia-news/europe-s-share-of-global-medicines-rd-shrinks-by-a-quarter-in-20-years-as-sector-s-declining-trends-continue/

7
https://ecipe.org/publications/strategic-autonomy-competitiveness-europes-innovative-pharmaceutical-sector/

8
https://www.ema.europa.eu/en/news/use-real-world-evidence-regulatory-decision-making-ema-publishes-review-its-studies

9
https://www.eahp.eu/sites/default/files/he_atmp_position_eptri_eahp_eueye_july2023_final_c.pdf?utm_medium=email&utm_campaign=website&utm_source=EAHP25Congress

10
https://www.bag.admin.ch/dam/bag/de/dokumente/biomed/transplantationsmedizin/studie-hospital-exemptions-atmp-eu-2022.pdf.download.pdf/studie-hospital-exemptions-atmp-eu-2022.pdf

## Abbreviations

ESOT, European Society for Organ Transplantation; ATMPs, Advanced Therapy Medicinal Products; EU, European Union; EMA, European Medicines Agency; HE, Hospital Exemption; RWE, Real-World Evidence; RWD, Real-World Data; EVOP, *Ex Vivo* Organ Perfusion; CAR, Chimeric Antigen Receptor; Treg, Regulatory T-cell; MSCs, Mesenchymal Stromal Cells; TRL, Technology Readiness Level; GMP, Good Manufacturing Practices; FDA, Food and Drug Administration; CAT, Committee for Advanced Therapies; EC, European Commission; ENVI, Committee on the Environment Public Health and Food Safety; EMA/CAT, EMA’s Committee for Advanced Therapies; SVF, Stromal Vascular Fraction; EC/1394/2000, Regulation EC/1394/2000; 2001/83/EC, Directive 2001/83/EC; FDA, Food and Drug Administration; GSK, GlaxoSmithKline.
